# Integrative Transcriptome-Wide Analyses Uncover Novel Risk-Associated MicroRNAs in Hormone-Dependent Cancers

**DOI:** 10.3389/fgene.2021.716236

**Published:** 2021-08-26

**Authors:** Dulari K. Jayarathna, Miguel E. Rentería, Adil Malik, Emilie Sauret, Jyotsna Batra, Neha S. Gandhi

**Affiliations:** ^1^Centre for Genomics and Personalised Health, School of Chemistry and Physics, Queensland University of Technology, Brisbane, QLD, Australia; ^2^Department of Genetics and Computational Biology, QIMR Berghofer Medical Research Institute, Brisbane, QLD, Australia; ^3^School of Biomedical Sciences, Queensland University of Technology, Brisbane, QLD, Australia; ^4^Translational Research Institute, Brisbane, QLD, Australia; ^5^School of Mechanical, Medical and Process Engineering, Queensland University of Technology, Brisbane, QLD, Australia

**Keywords:** hormones, microRNA, TWAS, SMR-HEIDI, pleiotropy

## Abstract

**Background:**

Hormone-dependent cancers (HDC) are among the leading causes of death worldwide among both men and women. Some of the established risk factors of HDC include unhealthy lifestyles, environmental factors, and genetic influences. Numerous studies have been conducted to understand gene–cancer associations. Transcriptome-wide association studies (TWAS) integrate data from genome-wide association studies (GWAS) and gene expression (expression quantitative trait loci – eQTL) to yield meaningful information on biological pathways associated with complex traits/diseases. Recently, TWAS have enabled the identification of novel associations between HDC risk and protein-coding genes.

**Methods:**

In the present study, we performed a TWAS analysis using the summary data-based Mendelian randomization (SMR)–heterogeneity in dependent instruments (HEIDI) method to identify microRNAs (miRNAs), a group of non-coding RNAs (ncRNAs) associated with HDC risk. We obtained eQTL and GWAS summary statistics from the ncRNA-eQTL database and the National Human Genome Research Institute–European Bioinformatics Institute (NHGRI-EBI) GWAS Catalog.

**Results:**

We identified 13 TWAS-significant miRNAs at *cis* regions (±1 Mb) associated with HDC risk (two, five, one, two, and three miRNAs for prostate, breast, ovarian, colorectal, and endometrial cancers, respectively). Among them, eight novel miRNAs were recognized in HDC risk. Eight protein-coding genes targeted by TWAS-identified miRNAs (*SIRT1*, *SOX4*, *RUNX2*, *FOXA1*, *ABL2*, *SUB1*, *HNRNPH1*, and *WAC*) are associated with HDC functions and signaling pathways.

**Conclusion:**

Overall, identifying risk-associated miRNAs across a group of related cancers may help to understand cancer biology and provide novel insights into cancer genetic mechanisms. This customized approach can be applied to identify significant miRNAs in any trait/disease of interest.

## Introduction

According to the World Health Organization (WHO), cancers cause one in six deaths worldwide, representing a major public health issue (Ferlay et al., 2015). Hormones such as testosterone, estrogen, and progesterone play an important role in the risk to multiple common cancers, such as prostate, breast, ovarian, endometrial, and colorectal (La Vecchia and Franceschi, 1991; Riman et al., 1998; Emons et al., 2000; Risbridger et al., 2010). Only hormones and their molecular functions cannot solely explain these hormone-dependent cancers (HDC). Other elements such as genetic variants, environmental factors (sunlight and ionizing radiation, organic and inorganic materials, viruses and bacteria, and air and water pollution) and unhealthy lifestyles also contribute to the biology of disease risk and progression.

Different analytical methods have been introduced to characterize the genetic mechanisms of these cancers. Genome-wide association studies (GWAS) have been successful in uncovering thousands of associations between genetic variants and different cancer types (Sud et al., 2017). However, these associations are often challenging to interpret and translate into knowledge about cancer biology because GWAS studies alone cannot provide functional evidence of causal genes mediated by GWAS-recognized single-nucleotide polymorphisms (SNPs; Visscher et al., 2017). This has motivated the development of new methods to prioritize causal genes at GWAS loci. Transcriptome-wide association studies (TWAS) integrate GWAS summary statistics with gene expression data (expression quantitative trait loci – eQTL) to discover gene expression–disease association (Wainberg et al., 2019). Early TWAS methods, such as PrediXcan, require individual-level GWAS and eQTL (Gamazon et al., 2015). Recent theoretical advancement has led to the use of GWAS and eQTL summary statistics, such as MetaXcan (Barbeira et al., 2016), FUSION (Gusev et al., 2016), and SMR (summary data-based Mendelian randomization)–HEIDI (heterogeneity in dependent instruments) (Zhu et al., 2016). Both MetaXcan and FUSION require expression weights from trained models using a gene expression reference panel along with GWAS summary statistics. In contrast, SMR–HEIDI considers only GWAS and eQTL summary statistics for efficient computing. SMR–HEIDI is a two-step analysis that can distinguish causal/pleiotropic associations from linkage models, whereas most other TWAS methods do not address this issue. Zhu et al. (2016) proposed the SMR–HEIDI theory using the principles of Mendelian randomization (MR). MR uses genetic variants as instrumental variables to provide information about the relationship between the causal effect of a (non-genetic) risk factor and the outcome of interest. MR analysis can reduce confounding and reverse causation and various biases that are associated with randomized controlled trials. Moreover, the SMR–HEIDI method can be applied for multiple-omics studies such as transcriptomics [messenger RNA (mRNA), ribosomal RNA, and non-coding RNA (ncRNA) such as microRNA (miRNA)], epigenomics [deoxyribonucleic acid (DNA) methylation and histone modification], metabolomics (metabolite levels), and proteomics (protein expression).

Previous HDC TWAS studies profiled a large set of protein-coding genes associated with genetic risk variants (Mortlock et al., 2020). In 2018, a large-scale TWAS on breast cancer was conducted using the MetaXcan method (Barbeira et al., 2016; Wu L. et al., 2018). The authors identified 48 genes at Bonferroni threshold of *p*-value < 5.82 × 10^–6^, including 14 genes: *ZSWIM5*, *LRRC3B*, *SPATA18*, *UBD*, *KLHDC10*, and *MIR31HG* (long non-coding), *RIC8A*, *B3GNT1*, and *RP11-867G23.10* (long non-coding), *RP11-218M22.1* (long non-coding), and *GALNT16*, *PLEKHD1*, *MAN2C1f*, and *CTD-2323K18.1f* (long non-coding) at loci not previously reported for breast cancer. Ferreira et al. (2019) integrated eQTL information across various tissues (adipose, breast, immune cells, spleen, and whole blood) with breast cancer GWAS results using EUGENE, conceptually similar to the PrediXcan method. The outcome of the study highlighted 88 genes as likely targets; among them, 26 were novel, and some of these novel genes (*HLF*, *PTPN22*, *RHBDD3*, and *IRF*) play a role in cancer cell elimination or inflammation. Moreover, 24 genes were found as likely targets of estrogen receptor-negative (ER-) risk variants (defined as the absence of ERs in breast cancer cells), and 11 were unique for ER- cases. In 2019, a large-scale TWAS on colorectal cancer using the SMR–HEIDI method identified four SNP loci – 11q23.1 [SNP located at locus 23.1 of the long arm (q) of chromosome 11], 3p21.1, 19q13.33, and 6p21.31 – responsible for colorectal cancer risk through the differential expression of three (*COLCA1*, *COLCA2*, and *C11orf53*), one (*SFMBT1*), one (*FUT1*), and one (class II *HLA*) gene transcripts, respectively (Law et al., 2019). In the same year, a TWAS study on high-grade serous epithelial ovarian cancer was conducted using FUSION (Gusev et al., 2019). The authors reported 25 candidate susceptibility genes of ovarian cancer and experimentally validated one of the genes, *CHMP4C*, by associating a variant that induces allele-specific exon inclusion. A large-scale TWAS study on prostate cancer identified 217 candidate susceptibility genes at 84 independent 1-Mb regions (Mancuso et al., 2018). The authors introduced a Bayesian probabilistic approach to prioritize genes at regions with multiple TWAS signals. The 90%-credible gene sets have been calculated, optimizing a maximum number of genes for a given credible set. Therefore, the list of 217 genes was reduced to 109 genes. The studies described above provide compelling evidence that TWAS could be successfully applied into HDC using protein-coding RNA expression (Mancuso et al., 2018; Wu L. et al., 2018; Ferreira et al., 2019; Gusev et al., 2019; Law et al., 2019).

TWAS applications on ncRNA transcriptomic data, such as miRNAs, remain elusive in literature. MiRNAs are small, endogenous, ncRNAs composed of 19–25 nucleotides in length (Bhaskaran and Mohan, 2014). Disruption of regulatory functions of miRNAs has been implicated in HDC etiology, making miRNAs promising biomarker and therapeutic candidates (Peng and Croce, 2016). The aim of this study is, therefore, to prioritize miRNAs at known risk regions of distinct HDC types by repurposing an existing TWAS approach, SMR–HEIDI.

In the present study, we identify causal or pleiotropic miRNA–HDC risk associations using the SMR–HEIDI method. In breast cancer, we extend the TWAS analysis for ER+ and ER- subtypes. Identifying biomarkers for ER subtypes would be beneficial in diagnosis, determining the risk of recurrence, and selecting treatment methods for breast cancer. In this study, the eQTL summary statistics were taken from the “ncRNA-eQTL,” in which original miRNA expression data was derived from the cohorts of The Cancer Genome Atlas (TCGA; Li et al., 2020). The GWAS summary statistics were downloaded from the datasets published in the National Human Genome Research Institute–European Bioinformatics Institute (NHGRI-EBI) GWAS catalog (Buniello et al., 2019). Two downstream analyses were carried out to identify TWAS-significant miRNAs that are involved in important pathways. First, TWAS-significant miRNA differential expression levels (between tumor and normal groups) were profiled using TCGA miRNA-seq expression data. Second, we predicted target genes for TWAS-significant miRNAs using three miRNA target prediction tools: miRDB, TargetScan 7.1, and miRTarBase (Agarwal et al., 2015; Chou et al., 2018; Chen and Wang, 2019). The intersecting results across three databases were considered as possible miRNA–target interactions. Cancer-related functions/pathways of predicted genes were found using the CancerMine database, the latest literature-mined database for cancer–gene associations (Lever et al., 2019). In conclusion, we used the statistical power of TWAS to identify the association of genetic risk and miRNA expression dysregulation and their target genes associated with complex diseases such as HDC.

## Materials and Methods

### Genome-Wide Association Studies Summary Statistics of HDC

The SMR–HEIDI method requires both GWAS and eQTL datasets from a similar population. Therefore, in this study, we collected data from European ancestry to satisfy one of the major assumptions of SMR–HEIDI analysis. The GWAS summary statistics of HDC were obtained from the NHGRI-EBI GWAS catalog (Buniello et al., 2019). The prostate cancer dataset was obtained from the latest GWAS study by Schumacher et al. (2018). The given study contains meta-analyzed genotype data from a custom high-density array of 46,939/27,910 (cases/controls) in prostate cancer from European ancestry, with previously genotyped data 32,255/33,202 also of European ancestry. We used the latest GWAS meta-analysis on breast cancer, consisting of 1,37,045/1,19,078 dominated by European ancestry (Michailidou et al., 2015). The breast cancer GWAS summary statistics are available for both overall and subgroups by ER status: ER + and ER-. The GWAS summary statistics of colorectal cancer were acquired from a recent study that included 4,562/3,82,756 participants of white British ancestry (Zhou et al., 2018). The endometrial cancer GWAS summary statistics were collected from a meta-GWAS analysis (accessed under the consent of the author) that used 12,906/1,08,976 samples of European ancestry (O’Mara et al., 2018). This cohort was taken from 17 studies identified *via* the Endometrial Cancer Association Consortium (ECAC), the Epidemiology of Endometrial Cancer Consortium (E2C2), and the UK Biobank. We gathered ovarian cancer GWAS summary statistics from a combined study which included the epithelial ovarian cancer GWAS study pooling data from multiple genome-wide genotyping projects totaling 25,509/40,941 of European ancestry (Phelan et al., 2017).

### MicroRNA-Expression Quantitative Trait Loci Summary Statistics

The miRNA eQTL summary statistics were retrieved from the ncRNA-eQTL database (Li et al., 2020). The ncRNA-eQTL database was developed using miRNA sequencing data and genotype data consisting of 8,734 TCGA samples. These samples are collected across 33 cancer types in European ancestry. In the ncRNA-eQTL pipeline, *cis*-eQTL, and *trans*-eQTL were identified by a computationally efficient analysis called matrix eQTL (Shabalin, 2012). In the matrix eQTL approach, significant eQTL SNPs are selected after adjusting for multiple testing using the Benjamini–Hochberg method, known as false discovery rate (FDR < 0.05) (Benjamini and Hochberg, 1995). *cis*-eQTL were defined when the SNP was within 1 Mb from the gene transcriptional start site and regulates the corresponding gene expression. In contrast, *trans*-eQTL were defined if the eQTL was beyond that region or on another chromosome. Here we used summary statistics for 7,463 (233 probes), 6,112 (286 probes), 1,651 (111 probes), 703 (50 probes), and 3,188 (177 probes) *cis*-eQTL SNPs from prostate, breast, ovarian, endometrial, and colorectal cancers, respectively, at a per-tissue FDR < 0.05. The *trans*-miRNA eQTL are not described as they failed to identify significant miRNAs in any HDC after HEIDI analysis. Both GWAS and eQTL SNPs were annotated using GRCh37 (hg19) to avoid misinterpretations. The “UCSC Genome Table Browser” was used for converting the SNP annotations (Karolchik et al., 2004).

### Transcriptome-Wide Association

**FIGURE 1:**
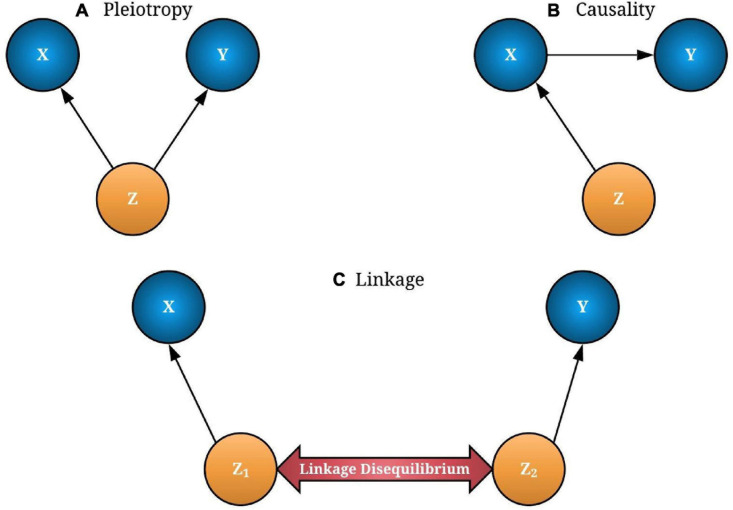
The possible associations between gene expression (X) and trait/disease (Y) through a causal variant single-nucleotide polymorphism (SNP-Z). If both X and Y are affected by SNP (Z), it is called “pleiotropy” **(A)**. If Y is affected by Z through X, it is known as causality **(B)**. In linkage models, two distinct causal variants (Z_1_ and Z_2_) can be in a linkage disequilibrium (LD) that causes X and Y independently **(C)**. Pleiotropy and causality of interest should be extracted from linkage models for a better transcriptome-wide association studies (TWAS) interpretation. The concept for the image was adapted from Zhu et al. (2016).

Using summary-level datasets for miRNA-eQTL and GWAS, we assessed the association between miRNA expression level and HDC risk using the SMR method, followed by a heterogeneity test – HEIDI (Zhu et al., 2016). The SMR applies the principle of MR theory and further described it in the SMR–HEIDI theoretical paper (Zhu et al., 2016). In SMR, the phenotypic trait is the outcome (Y), the expression level of a gene is the exposure (X), and the top associated *cis*-eQTL SNPs that are strongly associated with gene expression are used as an instrumental variable (*Z*) (*P*_eQTL_ < 5 × 10^–8^). The derived SMR test statistic (*T*_*SMR*_) is given in Equation 1.

(1) $$T_{\text{SMR}}=\left(z_{z\,y}^{2}\,z_{z\,x}^{2}\right)/\left(z_{z\,y}^{2}+z_{z\,x}^{2}\right)$$

where *Z*_*zy*_ and *Z*_*zx*_ are the *Z*-statistics from the GWAS and eQTL studies, respectively. In the SMR test, we used the FDR-adjusted *p*-value for multiple testing correction that provides a good balance between discovering statistically significant miRNAs and the limitation of false-positive occurrences (Benjamini and Hochberg, 1995). The significant miRNA–HDC risk associations were identified at 0.05 threshold. SMR introduces three models to describe the miRNA expression–trait association: pleiotropy (Z→X and Z→Y), causality (Z→X→Y), and linkage (Z_1_→X, Z_2_→Y), where Z_1_ and Z_2_ are two distinctive variants which are in a linkage disequilibrium (LD) within a *cis*-eQTL region (see Figure 1).

**FIGURE 2:**
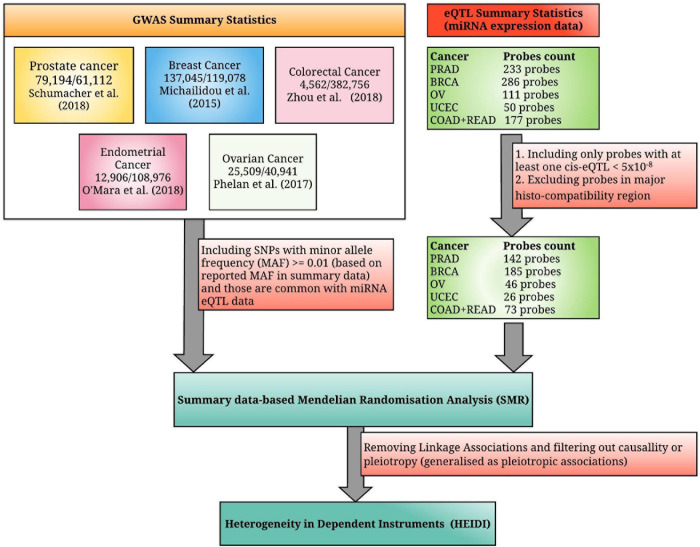
A summarized diagram for the analytical framework used in this study. We have integrated the genome-wide association studies (GWAS) summary statistics for the five most common hormone-dependent cancers (HDC) and microRNA expression quantitative trait loci (miRNA eQTL) summary statistics from the non-coding RNAs (ncRNA)-eQTL database, where the original miRNA expression data were collected from the miRNA-seq data of the Cancer Genome Atlas (TCGA). TCGA study names PRAD (prostate adenocarcinoma), BRCA (breast invasive carcinoma), OV (ovarian serous cystadenocarcinoma), UCEC (uterine corpus endometrial carcinoma), COAD (colon adenocarcinoma), and READ (rectum adenocarcinoma) denote prostate cancer, breast cancer, ovarian cancer, endometrial cancer, colon cancer, and rectal cancer, respectively (the combined miRNA eQTL data of colon and rectal cancers are considered as colorectal cancer eQTL data).

In Figures 1A,B, associations are considered as pleiotropic associations when a single genetic mutation affects both the miRNA expression and the trait of interest. The characterization of these pleiotropic mechanisms helps to explain the shared genetic architecture among different traits and diseases. A greater understanding of pleiotropy inevitably contributes to advances in precision medicine. Figure 1C illustrates a linkage association where the top-associated eQTL is in LD with two distinct causal variants, one affecting miRNA expression and the other affecting trait variation. These two linkage-associated causal variants are in less biological interest as they independently affect miRNA expression and the trait of interest. To exclude SMR results that may exhibit linkage, HEIDI test was introduced (Pavlides et al., 2016). Deriving HEIDI test statistic is available in the Methods section of Zhu et al. (2016). Recent SMR–HEIDI method-based studies have demonstrated that *P*_HEIDI_ > 0.01 provides better predictions compared to the *P*_HEIDI_ > 0.05 threshold defined in the original paper (Zhu et al., 2016; Wu Y. et al., 2018; Adolphe et al., 2021). Therefore, we used *P*_HEIDI_ > 0.01 to exclude miRNA genes that belong to the linkage models. At the interpretation of output, the positive/negative sign of SMR effect size, $\hat{b_{x\,y}}=\hat{b_{z\,y}}/\hat{\beta_{z\,x},}$where $\hat{b_{z\,y}}$is the estimate of a SNP effect from GWAS for a trait and $\hat{\beta_{z\,x}}$is the estimate of a SNP effect on the expression level of a gene from an independent eQTL study, has been utilized to predict the oncogenic/tumor suppressive role of miRNAs, respectively. Figure 2 illustrates the study design of our analysis.

### Profiling Expression Levels of HDC Risk-Associated miRNAs

Previous experimental and computational studies have shown that aberrant miRNA expression levels facilitate and abrogate the tumorigenic process. Our TWAS study identified significant miRNAs in HDC risk utilizing the expression of tumor tissues. We checked whether TWAS-identified miRNAs are differentially expressed between tumor and normal samples. The differential expression analysis was conducted using the DESeq2 R package (Love et al., 2014; R Development Core Team, 2017). The DESeq2 method tests for differential expression based on a model using the negative binomial distribution (Love et al., 2014). miRNA expression changes between tumor and normal tissues were visualized using MA plot (ggmaplot function in ggpubr R package^1^) (Wickam, 2016). The MA plot shows the log-transformed mean expression (A) on the *X*-axis and the log fold change (*M*) on the *Y*-axis. miRNAs with a similar expression between the normal and tumor groups will cluster around the *M* = 0 value, which means that miRNA expression does not exhibit a significant difference between two conditions: tumor and normal. If a miRNA is upregulated or downregulated, the point is above or below the *M* = 0 line, respectively. We used the genomic data commons (GDC) data portal to download the miRNA expression data reposited from HDC TCGA studies (Grossman et al., 2016).

### Identification of miRNA Target Genes

To identify miRNA target genes in likelihood, significant HDC risk-associated miRNAs were subjected to three miRNA target prediction tools: miRDB, TargetScan 7.1, and miRTarBase (Agarwal et al., 2015; Chou et al., 2018; Chen and Wang, 2019). The predicted genes were filtered based on two criteria recommended in previous studies: (i) 80 < miRDB score ≤ 100 and (ii) cumulative weighted context score (TargetScan 7.1) ≤ −0.2 (no lower bound) (Xue et al., 2018). Genes common among the three databases were selected and searched for cancer-associated literature using the CancerMine, a literature-mined database of drivers, oncogenes, and tumor suppressors in cancer (Lever et al., 2019). The genes that were significantly recorded in relevant HDC molecular functions/pathways are described in this work.

## Results

In our work, 13 miRNAs exhibited significant causal/pleiotropic associations with HDC risk (see Table 1).

**TABLE 1:** Summary data-based Mendelian randomization (SMR)–heterogeneity in dependent instruments (HEIDI) test results for significant microRNAs (miRNAs) in hormone-dependent cancers (HDC).

**Cancer type**	**Chr: base pair position (top SNP)**	**rs ID (top SNP)**	**Associated miRNA**	**Effect size**	**Standard error**	**FDR (SMR)**	***P*-value (HEIDI)**
Prostate	8:92060665	rs6999873	hsa-miR-4661-5p	−0.0898	0.0377	0.0174	0.2017
Prostate	9:73282815	rs10124022	hsa-miR-204-5p	−0.0517	0.0252	0.0401	0.8918
Breast	12: 54420098	rs4759318	hsa-miR-196a-3p	−0.0578	0.0221	0.0088	0.3310
Breast	2: 190013146	rs9288163	hsa-miR-3129-3p	−0.1011	0.0355	0.0044	0.1789
Breast	5: 148441321	rs36047	hsa-miR-584-5p	−0.1713	0.0564	0.0024	0.5121
Breast ER-	3: 195750742	rs9820939	hsa-miR-570-3p	−0.2441	0.0927	0.0085	0.3981
Breast ER+	2: 103068156	rs917998	hsa-miR-4772-5p	−0.0606	0.0253	0.0166	0.0547
Endometrial	11: 34894166	rs2915232	hsa-miR-1343-3p	0.0779	0.0373	0.0367	0.1240
Endometrial	1: 67088603	rs10789211	hsa-miR-3117-3p	0.0949	0.0432	0.0278	0.2559
Endometrial	2:219920412	rs3731881	hsa-miR-3131	−0.0933	0.0385	0.0155	0.0235
Ovarian	8: 8346690	rs2976909	hsa-miR-4660	−0.275	0.0987	0.0053	0.3963
Colorectal	2: 103066858	rs11465730	hsa-miR-4772-3p	0.1803	0.0809	0.0259	0.3620
Colorectal	2: 103034749	rs4851581	hsa-miR-4772-5p	0.0803	0.0414	0.0426	0.2020

*SMR, summary data-based Mendelian randomization; HEIDI, heterogeneity in dependent instruments; miRNAs, microRNAs; HDC, hormone-dependent cancers; Chr, chromosome number; SNP, single-nucleotide polymorphism; FDR, false discovery rate, adjusted *p*-value; ER, estrogen receptor; hsa, *Homo sapiens* (human organism); miR, mature microRNA; 3p, 3-prime; and 5p, 5-prime.*

The above-mentioned significant miRNA list was chosen by the FDR multiple testing correction method. The FDR method have improved the TWAS outcome compared to Bonferroni correction. The miRNAs significant from Bonferroni correction have been marked by an asterisk as described in Supplementary Tables 1–5. Among the listed 13 miRNAs, five miRNAs have been previously studied using computational and/or experimental methods (Li et al., 2010; Fils-Aimé et al., 2013; Liu et al., 2016; Lin et al., 2017; Yang et al., 2017). In contrast, the remaining novel set of eight miRNAs could be subjected to further study for their tumor-suppressive/oncogenic role in cancers. In the next subsections, we describe the TWAS results of each HDC in a detailed manner.

### Prostate Cancer

In prostate cancer eQTL data, 142 probes out of 233 were included in the analysis, with at least one *cis*-eQTL at *P*_eQTL_ < 5 × 10^–8^ (excluding probes in the major histocompatibility complex, MHC region). This probe exclusion criterion was followed for all HDC types to select strongly associated eQTLs with miRNA expression. In SMR, we identified six significant miRNAs (hsa-miR-22-5p, hsa-miR-5699-5p, hsa-miR-4661-5p, hsa-miR-155-5p, hsa-miR-194-3p, and hsa-miR-204-5p), where FDR < 0.05 (see Supplementary Table 1). After applying the HEIDI test, this reduced to two miRNAs, which are hsa-miR-4661-5p and hsa-miR-204-5p.

### Breast Cancer

We included 185 significant probes out of 286 into the breast cancer analysis. In breast cancer, three separate SMR tests were performed for the GWAS summary statistics from overall, ER+, and ER- analyses (see Supplementary Table 2). Nine miRNAs (hsa-miR-584-5p, hsa-miR-3129-3p, hsa-miR-196a-3p, hsa-miR-4746-5p, hsa-miR-548aw, hsa-miR-29a-5p, hsa-miR-944, hsa-miR-4766-3p, and hsa-miR-3615) passed the SMR analysis, and three of them (hsa-miR-584-5p, hsa-miR-3129-3p, and hsa-miR-196a-3p) were further selected from HEIDI analysis when miRNA eQTL are integrated with the overall GWAS summary statistics. In ER + and ER-, 13 (hsa-miR-944, hsa-miR-196a-3p, hsa-miR-4766-3p, hsa-miR-4772-5p, hsa-miR-3615, hsa-miR-425-3p, hsa-miR-101-3p, hsa-miR-3129-3p, hsa-miR-584-5p, hsa-miR-548aw, hsa-miR-6842-3p, hsa-miR-29a-5p, and hsa-miR-101-5p) and seven (hsa-miR-584-5p, hsa-miR-570-3p, hsa-miR-376b-5p, hsa-miR-376c-5p, hsa-miR-3129-3p, hsa-miR-328-3p, and hsa-miR-4781-3p) miRNAs passed the SMR analysis, respectively. These miRNAs were further analyzed by the HEIDI test to avoid linkage associations. Four and three miRNAs from the ER + and ER- groups, respectively, remained significant after the HEIDI test. We observed that hsa-miR-584-5p, hsa-miR-196a-3p, and hsa-miR-3129-3p were common to both overall and ER + groups. The hsa-miR-4772-5p was significant only in the ER + group. The hsa-miR-584-5p and hsa-miR-3129-3p were common in both the overall GWAS and ER- groups. The hsa-miR-570-3p was found to be significant only in ER- breast cancer.

### Endometrial Cancer

In endometrial cancer, 26 probes were chosen from 50 probes. We identified three miRNAs (hsa-miR-1343-3p, hsa-miR-3117-3p, and hsa-miR-3131) from the SMR test, and all of them were recognized as pleiotropically significant by HEIDI analysis (see Supplementary Table 3).

### Ovarian Cancer

We included 46 probes out of 111 for the SMR analysis. There were three significant probe–trait associations (hsa-miR-4660, hsa-miR-4758-5p, and hsa-miR-576-5p) that were selected from the SMR test, and they were further analyzed by the HEIDI method (see Supplementary Table 4). We identified hsa-miR-4660 as the only pleiotropic (non-linkage) miRNA from the HEIDI test.

### Colorectal Cancer

Herein we have integrated colon (152 probes) and rectal (25 probes) cancer eQTL to prepare the eQTL summary statistics of colorectal cancer. There were 73 probes in the SMR analysis after excluding low-expressed and MHC region probes. Among them, seven miRNA eQTLs (hsa-miR-144-5p, hsa-miR-144-3p, hsa-miR-153-5p, hsa-miR-1228-3p, hsa-miR-4772-3p, hsa-miR-3651, and hsa-miR-4772-5p) passed the SMR test, and two of them, hsa-miR-4772-3p and hsa-miR-4772-5p, passed the HEIDI test (see Supplementary Table 5). We observed that both HEIDI-significant miRNAs originate from the same family, hsa-miR-4772. One of them, hsa-miR-4772-5p, was also observed as significant in ER + breast cancer.

### Profiling Expression of HDC Risk-Associated miRNAs

**FIGURE 3:**
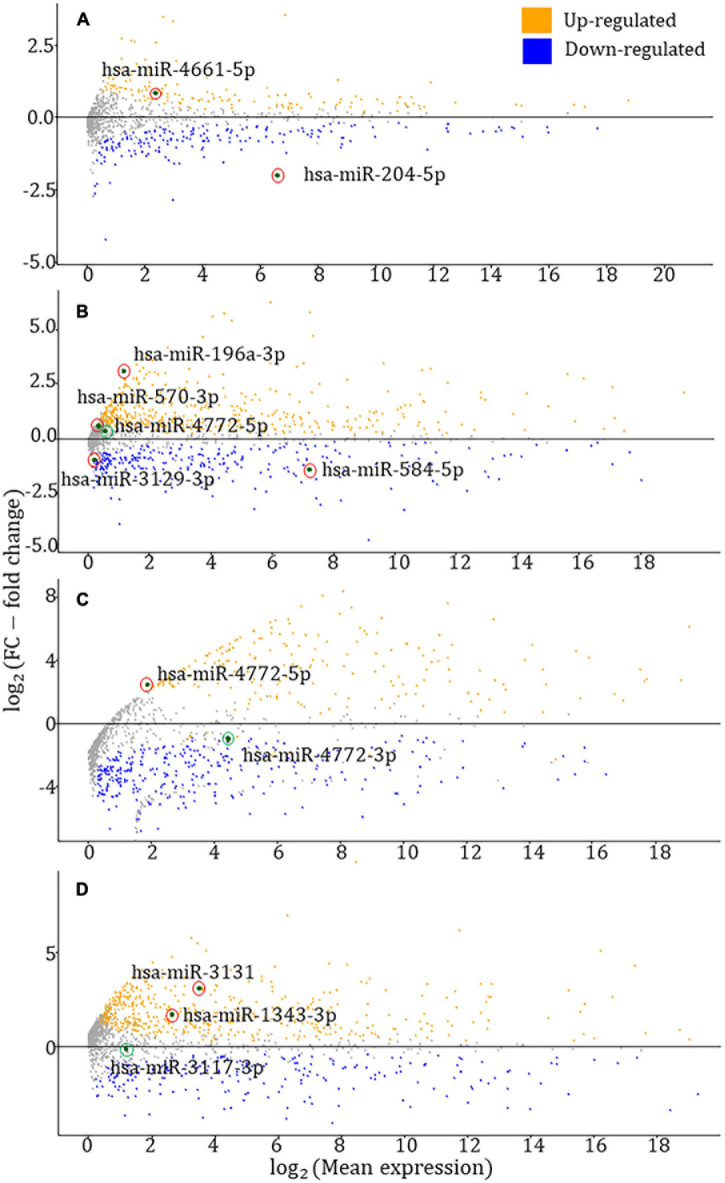
MA plots for the differential expression analysis of TWAS-identified miRNAs. **(A–D)** MA plots for prostate, breast, colorectal, and endometrial cancers, respectively. The red and green circled data points represent statistically significant [false discovery rate (FDR) < 0.05)] and insignificant (FDR ≥ 0.05) miRNAs from the DESeq2 differential expression analysis (Love et al., 2014). According to the four figures in the panel, six miRNAs have shown upregulations, whereas three miRNAs have exhibited downregulation.

Aberrant miRNA expression profiles may heavily impact the development, differentiation, and control of growth, leading to cancers. Therefore, profiling the expression of identified HDC risk-associated miRNAs will strengthen our TWAS outcome. For each HDC, TWAS-identified miRNAs were contrasted between tumor *vs*. normal samples at FDR < 0.05 threshold. MA plots were drawn to visualize the differential expression analyses results. In MA plots in Figure 3, red- and green-circled points denote statistically significant (FDR < 0.05) and insignificant (FDR ≥ 0.05) miRNAs, respectively.

As reported by the differential expression analyses, the FDR-adjusted *p*-values of hsa-miR-204-5p, hsa-miR-4661-5p, hsa-miR-196a-3p, hsa-miR-570-3p, hsa-miR-584-5p, hsa-miR-3129-3p, hsa-miR-4772-5p (BRCA – breast invasive carcinoma), hsa-miR-4772-3p (COAD – colon adenocarcinoma), hsa-miR-4772-5p (COAD), hsa-miR-1343-3p, hsa-miR-3117-3p, and hsa-miR-3131 are 1.14 × 10^–40^, 8.07 × 10^–6^, 1.01 × 10^–21^, 2.48 × 10^–2^, 1.47 × 10^–25^, 6.41 × 10^–9^, 2.30 × 10^–1^, 8.67 × 10^–2^, 2.07 × 10^–2^, 8.49 × 10^–12^, 9.54 × 10^–1^, and 2.05 × 10^–7^, respectively. The miRNA expression data were collected from the relevant HDC study of TCGA – for instance, hsa-miR-204-5p expression data were gathered from the prostate cancer study of TCGA. In the colorectal cancer-associated MA plot, the most significant adjusted *p*-values were chosen among COAD and READ (rectum adenocarcinoma) studies.

### Identifying HDC Risk-Associated miRNA Target Genes

The miRNA target genes that appear common (shared) among miRDB, miRTarBase, and TargetScan 7.1 are presented in Table 2. The target genes that have strong evidence with relevant HDC-associated studies (highlighted in Table 2) will be explained in the section “Discussion.”

**TABLE 2:** Shared miRNA–gene interactions among miRDB, TargetScan, and miRTarBase for the transcriptome-wide association studies (TWAS)-identified miRNAs.

**hsa-miR-204-5p**		**hsa-miR-4661-5p**	**hsa-miR-196a-3p**	**hsa-miR-570-3p**	**hsa-miR-584-5p**	**hsa-miR-3129-3p**	**hsa-miR-4772-5p**	**hsa-miR-4772-3p**	**hsa-miR-1343-3p**	**hsa-miR-3117-3p**	**hsa-miR-3131**	**hsa-miR-4660**
*AKAP1*	*JAK2*	None	*SEC31A*	***ABL2***	*ELL2*	*SLC35A5*	***HNRNPH1***	*RBM48*	*AGTPBP1*	*AZI2*	*RCC2*	*HERPUD2*
*ANKRD13A*	*JARID2*			*BAG4*	***FOXA1***		*NCKAP1*		*ATXN7L3*			*NPTXR*
*AP1S2*	*MAP1LC3B*			*C18orf25*	*GNG12*		***WAC***		*CBX5*			*SCAMP2*
*BMPR1A*	*MAPRE2*			*CCDC117*	*MORC3*				*LARP1*			
*CCNT2*	*RAB22A*			*CHSY1*	*PPIL1*				*TGFBR1*			
*CDH4*	***RUNX2***			*GNA13*	*ZNF121*				*ZNF704*			
*CHD5*	*SAMD5*			*KCNB1*								
*CREB5*	***SIRT1***			*PHF6*								
*EZR*	*SLC43A1*			*SAMD12*								
*FOXC1*	***SOX4***			***SUB1***								
*GAN*	*TCF12*			*TRIP13*								
*HAS2*	*TGFBR2*			*TSC22D2*								
*HCAR2*	*WWC3*											
*HOXC8*	*ZCCHC24*											
*IGFBP5*												

*Columns represent microRNAs (miRNAs) identified from the TWAS study. Rows represent target genes (shared in all three miRNA target databases – miRDB, TargetScan, and miRTarBase) for each miRNA.*

*The bolded words represent target genes that have shown strong evidence with relevant HDC-associated studies.*

## Discussion

Our study is the first TWAS-level study in identifying miRNAs in multiple hormonal cancers. Herein each TWAS analysis was followed by two downstream analyses: (i) tumor-control expression profiling analysis (differential expression analysis and MA plot) and (ii) investigation of tumor-suppressive/oncogenic role of miRNA-targeted genes from the literature curated databases. Using SMR, we initially identified 38 miRNA–HDC risk associations, and 13 of them were selected by a subsequent HEIDI test after removing linkage associations.

Six significant miRNAs were observed in prostate cancer analysis, and only two of them passed the HEIDI test, hsa-miR-204-5p and hsa-miR-4661-5p. In both miRNAs, the SMR effect sizes’ signs are negative, predicting them as tumor-suppressive miRNAs. Previous studies confirmed that miR-204-5p promotes apoptosis by targeting *BCL2* in prostate cancer cells, validating its tumor-suppressive role (Lin et al., 2017). The role of hsa-miR-4661-5p in the tumor environment has not been reported in the literature to date. According to MA plots drawn by differential expression analysis, hsa-miR-204-5p and hsa-miR-4661-5p have been downregulated and upregulated in prostate cancer, respectively. The downregulation of hsa-miR-204-5p supports for its tumor-suppressive role described in previous experimental studies (Lin et al., 2017). We found 29 target genes for hsa-miR-204-5p that were recorded in miRDB, miRTarBase, and TargetScan 7.1, and none of the hsa-miR-4661-5p target genes were common among these three databases. Among these hsa-miR-204-5p target genes, *SIRT1*, *SOX4*, and *RUNX2* exhibited a tumor-suppressive/oncogenic role in previous prostate cancer studies. *SIRT1* is involved in androgen-mediated transcriptional repression and growth suppression of prostate cancer cells (Dai et al., 2007). The *SOX4* gene has been identified as a critical component of the PTEN-PI3K-AKT pathway in prostate cancer (Bilir et al., 2016), and *RUNX2* is a type of oncogene that unusually increases in prostate cancer cells, promoting their metastatic phenotype (Baniwal et al., 2010). Further experimental work is required to understand the combined effect of hsa-miR-204-5p and given target genes in prostate cancer pathways and networks.

Recently, Larson et al. (2020) performed a miRNA-based TWAS analysis of prostate cancer using the TWAS–FUSION method. The authors reported two (miR-941 family and miR-3617-5p) and one (hsa-miR-16-2-3p) significant miRNA from normal and tumor expression models, respectively. None of these miRNAs were found in our study. The absence of common miRNAs between the study of Larson et al. (2020) and ours can be due to applying different statistical approaches over different populations. The miRNAs found from the above-mentioned study have previously been reported as having tumor-suppressive/oncogenic properties in other cancers, but not in prostate cancer.

We performed the SMR–HEIDI test for three GWAS summary statistics datasets of breast cancer. Three miRNAs were significant – hsa-miR-196a-3p, hsa-miR-584-5p, and hsa-miR-3129-3p – when the overall GWAS summary statistics were applied in the SMR–HEIDI analysis and predicted as tumor-suppressive by their negative effect sizes. Among them, hsa-miR-196a-3p and hsa-miR-584-5p were previously reported as tumor suppressors in breast cancer. Three members of the miR-196 family where the resulting miR-196a-3p belongs – miR-196a-1, miR-196a-2, and miR-196b – could suppress breast cancer cell migration and metastasis by inhibiting *HOXC8*, which promotes tumorigenesis by regulating the expression of cadherin-11 in breast cancer (Li et al., 2010). miR-584 has been identified as a novel target of TGF-beta that plays a role in breast cancer progression as a prometastatic factor (Fils-Aimé et al., 2013). The upregulation of hsa-miR-3129 is known to suppress epithelial ovarian cancer through *CD44*. The *CD44* gene is highly expressed in many cancers and regulates metastasis (Sun et al., 2018). In the SMR–HEIDI analysis of ER + and ER- breast cancer subgroups, hsa-miR-4772-5p and hsa-miR-570-3p were recognized as tumor-suppressive miRNAs, respectively. Further experiments are required to establish the role of hsa-miR-4772-5p and hsa-miR-570-3p in breast cancer. In our study, hsa-miR-4772-5p was TWAS significant in both breast (ER+) and colorectal cancer risk.

As reported by the differential expression analysis of breast cancer, hsa-miR-584-5p and hsa-miR-3129-3p have shown a statistically significant downregulation in breast cancer. The tumor-suppressive role of hsa-miR-584-5p described in literature is supported by observing its downregulation in our differential expression analysis (Fils-Aimé et al., 2013). Two miRNAs – hsa-miR-196a-3p and hsa-miR-570-3p – have shown upregulation in breast cancer. Among five TWAS-identified miRNAs, hsa-miR-4772-5p did not exhibit a statistically significant expression level difference between breast tumor and normal samples. The target genes of five miRNAs were searched across three miRNA–gene target prediction tools – miRDB, TargetScan 7.1, and miRTarBase. We found six, one, one, 12, and three possible target genes for hsa-miR-584-5p, hsa-miR-196a-3p, hsa-miR-3129-3p, hsa-miR-570-3p, and hsa-miR-4772-5p, respectively. One gene targeted by hsa-miR-584-5p – *FOXA1* – and two genes targeted by hsa-miR-570-3p – *ABL2* and *SUB1* – have been described in previous breast cancer studies. *FOXA1* positively regulates gene expression by altering the gene methylation status in human breast cancer Michigan Cancer Foundation-7 (MCF-7) cells (Zheng et al., 2015). Knocking down of *ABL2* in breast cancer cells (using a mouse xenograft model) leads to increased tumor cell proliferation and a significantly enlarged tumor size in breast cancer (Gil-Henn et al., 2013). The *SUB1* gene can promote breast cancer proliferation and metastasis through the c-Myc-mediated Warburg effect (Luo et al., 2019).

In colorectal cancer, hsa-miR-4772-3p and hsa-miR-4772-5p were significant from TWAS analyses, and both originate from hsa-miR-4772 precursor miRNA. These two miRNAs could be predicted as oncogenic by their positive SMR effect sizes. Previous studies have shown that the under-expression of serum exosomal hsa-miR-4772-3p could discriminate colon cancer recurrence patients from non-recurrence (Liu et al., 2016). Thus, it might serve as a prognostic biomarker for colon cancer patients with tumor recurrence. hsa-miR-4772-5p has been differentially expressed in *Fusobacterium nucleatum*, which increases the proliferation of colorectal cancer cells and tumor development (Yang et al., 2017). Conforming to differential expression analysis, hsa-miR-4772-5p has shown a statistically significant upregulation, whereas hsa-miR-4772-3p was insignificant. Across the three miRNA–gene target prediction tools, we found three and one gene targeted by hsa-miR-4772-5p and hsa-miR-4772-3p, respectively. Among them, *HNRNPH1* and *WAC*, targeted by hsa-miR-4772-5p, have been described in previous colorectal cancer studies. A recent study has shown that the *HNRNPH1*-induced stabilization of SGPL1 mRNA promoted tumor progression by inhibiting p53 phosphorylation in colorectal cancer cells (Takahashi et al., 2020). The *WAC* gene has been mutated in murine colorectal cancer mutagenesis screens, and that reduction in *WAC* expression reduces cell growth (Clark et al., 2016).

Three significant miRNAs in endometrial cancer – hsa-miR-1343-3p, hsa-miR-3117-3p, and hsa-miR-3131 – were not recorded in previous endometrial cancer studies. Among them, two miRNAs – hsa-miR-1343-3p and hsa-miR-3117-3p – and one miRNA – hsa-miR-3131 – can be predicted as oncogenes and a tumor suppressor, due to positive and negative SMR effect sizes, respectively. The differential expression analysis has shown that both hsa-miR-1343-3p and hsa-miR-3131 have been upregulated in endometrial cancer. One TWAS-identified miRNA in endometrial cancer – hsa-miR-3117-3p – did not show a statistically significant expression level difference between the tumor and normal cohorts. The three prediction tools provided six, one, and one target gene for hsa-miR-1343-3p, hsa-miR-3117-3p, and hsa-miR-3131, respectively. None of these target genes were reported in endometrial cancer literature. In ovarian cancer, hsa-miR-4660 was identified as a novel risk-associated miRNA that can be predicted as an onco-suppressor due to its negative SMR effect size. We could not perform a differential expression analysis of ovarian cancer as normal cohort data was not accessible from TCGA. We found three target genes for hsa-miR-4660, and none of these genes have been reported in past ovarian cancer studies. Both endometrial and ovarian cancer TWAS/SMR–HEIDI analyses resulted in four novel miRNAs into the stream of cancer studies that could be further validated using cancer-associated miRNA functional experiments.

We acknowledged that our approach has certain limitations, and these apply equally to the application of SMR–HEIDI in an epidemiological context. In this work, the sample sizes are limited (472 *cis*-eQTL probes) compared to the protein-coding eQTL summary statistics (most are in thousands/ten thousands scale). This minimizes our TWAS results compared to that of the large-scale protein-coding gene expression analyses. This SMR–HEIDI method has assumed the existence of a single causal variant per locus. It is possible to find multiple causal SNPs for a given locus according to the recent theory of allelic heterogeneity (Jansen et al., 2017). An alternative method, the generalized SMR (GSMR), is available for multi-SNP MR analysis. GSMR cannot be used in this work as it requires summary-level data only from GWAS studies (Zhu et al., 2018).

Our study did not consider the effect of distal SNPs and mediating biomarkers, such as CpG sites, copy number alteration, and transcription factors (Bhattacharya et al., 2021). In HEIDI analysis, a few SMR associations were not considered as their top-associated *cis*-eQTL had only one or two SNPs in the *cis* region. Furthermore, the HEIDI method cannot distinguish causality from pleiotropy. We had a limited set of target genes for HDC risk-associated miRNAs at miRNA target predictions in downstream analyses. The functions of these miRNAs are not/less reported in previous experimental/computational work. Therefore, the number of target genes identified across three prediction tools was inadequate for a functional enrichment analysis/protein–protein interaction analysis. Despite these caveats, our findings shed a new light on the role of miRNAs in HDC risk using a customized TWAS approach.

In summary, we repurposed an existing TWAS approach, SMR–HEIDI, to analyze miRNA expression data. We identified 13 miRNAs (miRNA SNPs) that are associated with HDC risk. Among them, one (hsa-miR-4661-5p), three (hsa-miR-3129-3p, hsa-miR-570-3p, and hsa-miR-4772-5p), one (hsa-miR-4660), and three (hsa-miR-1343-3p, hsa-miR-3117-3p, and hsa-miR-3131) miRNAs were novel for prostate, breast, ovarian, and endometrial cancers, respectively. The differential expression analysis of TWAS-identified miRNAs has shown that 9 out of 13 miRNAs are differentially expressed in HD tumor tissues of interest. Some of the protein-coding genes targeted by TWAS-identified miRNAs have been reported as tumor repressors or oncogenes in previous HDC studies. These observations provide confidence to our statistical miRNA predictions. This study has used miRNA eQTL information from tumor tissues only. In a future work, we will focus the TWAS analyses on identifying miRNA–HDC risk associations using miRNA eQTL generated from normal tissues subject to the availability of datasets. The set of HDC risk-associated miRNAs found in this study needs to be functionally characterized further and may potentially be utilized to develop biomarkers and therapeutic drug designs. Importantly, the same analytical approach can be implemented to detect associations between miRNA expression and other traits of interest.

## Conclusion

We customized an existing TWAS approach to identify risk-associated miRNAs in HDC types. Our approach prioritized 13 miRNAs associated with individual HDC, and such a method could be extended to study other complex traits/diseases. The putative miRNAs and their target genes identified in our study will enable us to understand the HDC biology better. This study was conducted utilizing a limited number of miRNA eQTL originated from European ancestry-based genetic datasets. Future miRNA-based TWAS analyses are warranted for diverse datasets generated from different populations/ancestries.

## Data Availability Statement

Publicly available datasets were analyzed in this study (except endometrial cancer GWAS summary statistics). These data can be found here: the NHGRI-EBI GWAS Catalog (https://www.ebi.ac.uk/gwas/), the ncRNA-eQTL database (http://ibi.hzau.edu.cn/ncRNA-eQTL/), and TCGA HDC miRNA expression from the National Cancer Institute GDC Data Portal (https://portal.gdc.cancer.gov/repository). TWAS analyses were conducted using the SMR-HEIDI version 1.03, freely available at https://cnsgenomics.com/software/smr/#Download.

## Author Contributions

DJ contributed to the conceptualization and methodology, performed statistical analyses, interpreted results, participated in writing — original draft, writing — review, and editing. NG and MR contributed to the conceptualization and methodology and participated in writing – review and editing. JB contributed to the conceptualization and participated in writing — review and editing. ES participated in writing — review and editing. AM contributed to the methodology. All the authors read and approved the final manuscript.

## Conflict of Interest

The authors declare that the research was conducted in the absence of any commercial or financial relationships that could be construed as a potential conflict of interest.

## Publisher’s Note

All claims expressed in this article are solely those of the authors and do not necessarily represent those of their affiliated organizations, or those of the publisher, the editors and the reviewers. Any product that may be evaluated in this article, or claim that may be made by its manufacturer, is not guaranteed or endorsed by the publisher.

## Footnotes

1
https://rpkgs.datanovia.com/ggpubr

## Abbreviations

BRCA: breast invasive carcinoma
ceRNA: competing endogenous RNA
COAD: colon adenocarcinoma
DNA: deoxyribonucleic acid
E2C2: Epidemiology of Endometrial Cancer Consortium
ECAC: Endometrial Cancer Association Consortium
ER: estrogen receptor
FDR: false discovery rate
GDC: genomic data commons
GWAS: genome-wide association studies
HDC: hormone-dependent cancers
HEIDI: heterogeneity in dependent instruments
LD: linkage disequilibrium
MCF-7: Michigan Cancer Foundation-7
MHC: major histocompatibility complex
miRNA: microRNA
mRNA: messenger RNA
NHGRI-EBI: National Human Genome Research Institute–European Bioinformatics Institute
OV: ovarian serous cystadenocarcinoma
PRAD: prostate adenocarcinoma
READ: rectal adenocarcinoma
RNA: ribonucleic acid
SMR: summary data-based Mendelian randomization
SNP: single-nucleotide polymorphism
TCGA: the Cancer Genome Atlas
TWAS: transcriptome-wide association studies
UCEC: uterine corpus endometrial carcinoma
WHO: World Health Organization.
