# Supplementary material for: Integrative Transcriptome-Wide Analyses Uncover Novel Risk-Associated MicroRNAs in Hormone-Dependent Cancers
Source: Front Genet. 2021 Aug 26;12:716236. doi: 10.3389/fgene.2021.716236 (PMC8427606; doi:10.3389/fgene.2021.716236)
Supplement: Supplementary file 1 [file Table_1.docx]

Table S1. SMR-HEIDI test results of prostate cancer

| Cancer type | Chr: base pair position (top SNP) | rs ID (top SNP) | Associated miRNA | Effect Size | Standard Error | FDR (SMR) | P-value (HEIDI) |
| --- | --- | --- | --- | --- | --- | --- | --- |
| Prostate | 17:618965 | rs684232 | hsa-miR-22-5p* | -0.5200 | 0.1426 | 0.0003 | NA |
| Prostate | 10:991737 | rs10904588 | hsa-miR-5699-5p | -0.1511 | 0.0615 | 0.0141 | NA |
| Prostate | 8:92060665 | rs6999873 | hsa-miR-4661-5p | -0.0898 | 0.0377 | 0.0174 | 0.2017 |
| Prostate | 21:26530598 | rs2829580 | hsa-miR-155-5p | 0.1625 | 0.0707 | 0.0216 | NA |
| Prostate | 11:65179436 | rs3741389 | hsa-miR-194-3p | 0.0932 | 0.0449 | 0.0377 | NA |
| Prostate | 9:73282815 | rs10124022 | hsa-miR-204-5p | -0.0517 | 0.0252 | 0.0401 | 0.8918 |
| SMR, summary data-based Mendelian randomisation; HEIDI, heterogeneity in dependent instruments; Chr, chromosome number; SNP, single nucleotide polymorphism; FDR, false discovery ratio, adjusted p-value; hsa, homo sapiens (human organism); miR, mature microRNA; 3p, 3-prime; 5p, 5-prime; NA reports if the number of SNPs used in the HEIDI analysis is smaller than 3. | | | | | | | |
