# Supplementary material for: Integrative Transcriptome-Wide Analyses Uncover Novel Risk-Associated MicroRNAs in Hormone-Dependent Cancers
Source: Front Genet. 2021 Aug 26;12:716236. doi: 10.3389/fgene.2021.716236 (PMC8427606; doi:10.3389/fgene.2021.716236)
Supplement: Supplementary file 2 [file Table_2.docx]

Table S2. SMR-HEIDI test results of breast cancer and ER sub-groups

| Cancer type | Chr: base pair position (top SNP) | rs ID (top SNP) | Associated miRNA | Effect Size | Standard Error | FDR (SMR) | P-value (HEIDI) |
| --- | --- | --- | --- | --- | --- | --- | --- |
| Breast-Overall | 5:148441321 | rs36047 | hsa-miR-584-5p* | -0.1713 | 0.0564 | 0.0024 | 0.5121 |
| Breast-Overall | 2:190013146 | rs9288163 | hsa-miR-3129-3p | -0.1011 | 0.0355 | 0.0044 | 0.1789 |
| Breast-Overall | 12:54420098 | rs4759318 | hsa-miR-196a-3p | -0.0578 | 0.0221 | 0.0088 | 0.3310 |
| Breast-Overall | 19:4445073 | rs2705 | hsa-miR-4746-5p | -0.1086 | 0.0512 | 0.0339 | NA |
| Breast-Overall | 9:135822290 | rs7035308 | hsa-miR-548aw | -0.1238 | 0.0591 | 0.0363 | NA |
| Breast-Overall | 7:130651614 | rs17165310 | hsa-miR-29a-5p | 0.3336 | 0.1324 | 0.0118 | NA |
| Breast-Overall | 3:189802871 | rs7633358 | hsa-miR-944 | -0.2065 | 0.0791 | 0.0091 | NA |
| Breast-Overall | 22:40264551 | rs5995786 | hsa-miR-4766-3p | -0.2082 | 0.0933 | 0.0256 | NA |
| Breast-Overall | 17:72767435 | rs880827 | hsa-miR-3615 | 0.1446 | 0.0586 | 0.0135 | NA |
| Breast-ER+ | 3:189802871 | rs7633358 | hsa-miR-944 | -0.2329 | 0.0916 | 0.0110 | NA |
| Breast-ER+ | 12:54420098 | rs4759318 | hsa-miR-196a-3p | -0.0675 | 0.0266 | 0.0112 | 0.4053 |
| Breast-ER+ | 22:40264551 | rs5995786 | hsa-miR-4766-3p | -0.2900 | 0.1203 | 0.0159 | NA |
| Breast-ER+ | 2:103068156 | rs917998 | hsa-miR-4772-5p | -0.0606 | 0.0253 | 0.0166 | 0.0547 |
| Breast-ER+ | 17:72767435 | rs880827 | hsa-miR-3615 | 0.1569 | 0.0685 | 0.0219 | NA |
| Breast-ER+ | 3:49751585 | rs2291542 | hsa-miR-425-3p | -0.2460 | 0.1082 | 0.0230 | NA |
| Breast-ER+ | 1:66410541 | rs11208775 | hsa-miR-101-3p | 0.2838 | 0.1293 | 0.0282 | NA |
| Breast-ER+ | 2:190013146 | rs9288163 | hsa-miR-3129-3p | -0.0889 | 0.0407 | 0.0290 | 0.1740 |
| Breast-ER+ | 5:148441321 | rs36047 | hsa-miR-584-5p | -0.1280 | 0.0589 | 0.0297 | 0.7890 |
| Breast-ER+ | 9:135822290 | rs7035308 | hsa-miR-548aw | -0.1569 | 0.0726 | 0.0307 | NA |
| Breast-ER+ | 8:28218893 | rs2294109 | hsa-miR-6842-3p | -0.5550 | 0.2626 | 0.0346 | NA |
| Breast-ER+ | 7:130651614 | rs17165310 | hsa-miR-29a-5p | 0.2629 | 0.1281 | 0.0401 | NA |
| Breast-ER+ | 1:66144876 | rs10158937 | hsa-miR-101-5p | 0.1600 | 0.0814 | 0.0493 | NA |
| Breast-ER- | 5:148441321 | rs36047 | hsa-miR-584-5p | -0.2680 | 0.0971 | 0.0058 | 0.2701 |
| Breast-ER- | 3:195750742 | rs9820939 | hsa-miR-570-3p | -0.2441 | 0.0927 | 0.0085 | 0.3981 |
| Breast-ER- | 14:101739680 | rs8008153 | hsa-miR-376b-5p | -0.2936 | 0.1349 | 0.0295 | NA |
| Breast-ER- | 14:101739680 | rs8008153 | hsa-miR-376c-5p | -0.2936 | 0.1360 | 0.0308 | NA |
| Breast-ER- | 2:190013146 | rs9288163 | hsa-miR-3129-3p | -0.1269 | 0.0620 | 0.0407 | 0.4624 |
| Breast-ER- | 16:68185160 | rs7193701 | hsa-miR-328-3p | 0.5443 | 0.2706 | 0.0442 | NA |
| Breast-ER- | 1:54515041 | rs10888832 | hsa-miR-4781-3p | 0.2550 | 0.1300 | 0.0498 | NA |

SMR, summary data-based Mendelian randomisation; HEIDI, heterogeneity in dependent instruments; ER, oestrogen-receptor; Chr, chromosome number; SNP, single nucleotide polymorphism; FDR, false discovery ratio, adjusted p-value; hsa, homo sapiens (human organism); miR, mature microRNA; 3p, 3-prime; 5p, 5-prime; NA reports if the number of SNPs used in the HEIDI analysis is smaller than 3.
