# Supplementary material for: Integrative Transcriptome-Wide Analyses Uncover Novel Risk-Associated MicroRNAs in Hormone-Dependent Cancers
Source: Front Genet. 2021 Aug 26;12:716236. doi: 10.3389/fgene.2021.716236 (PMC8427606; doi:10.3389/fgene.2021.716236)
Supplement: Supplementary file 3 [file Table_3.docx]

Table S3. SMR-HEIDI test results of endometrial cancer

| Cancer type | Chr: base pair position (top SNP) | rs ID (top SNP) | Associated miRNA | Effect Size | Standard Error | FDR (SMR) | P-value (HEIDI) |
| --- | --- | --- | --- | --- | --- | --- | --- |
| Endometrial | 2:219920412 | rs3731881 | hsa-miR-3131* | -0.0933 | 0.0385 | 0.0155 | 0.0235 |
| Endometrial | 1:67088603 | rs10789211 | hsa-miR-3117-3p | 0.0949 | 0.0432 | 0.0278 | 0.2559 |
| Endometrial | 11:34894166 | rs2915232 | hsa-miR-1343-3p | 0.0779 | 0.0373 | 0.0367 | 0.1240 |

SMR, summary data-based Mendelian randomisation; HEIDI, heterogeneity in dependent instruments; Chr, chromosome number; SNP, single nucleotide polymorphism; FDR, false discovery ratio, adjusted p-value; hsa, homo sapiens (human organism); miR, mature microRNA; 3p, 3-prime; 5p, 5-prime; NA reports if the number of SNPs used in the HEIDI analysis is smaller than 3.
