# Supplementary material for: Integrative Transcriptome-Wide Analyses Uncover Novel Risk-Associated MicroRNAs in Hormone-Dependent Cancers
Source: Front Genet. 2021 Aug 26;12:716236. doi: 10.3389/fgene.2021.716236 (PMC8427606; doi:10.3389/fgene.2021.716236)
Supplement: Supplementary file 4 [file Table_4.docx]

Table S4. SMR-HEIDI test results of ovarian cancer

| Cancer type | Chr: base pair position (top SNP) | rs ID (top SNP) | Associated miRNA | Effect Size | Standard Error | FDR (SMR) | P-value (HEIDI) |
| --- | --- | --- | --- | --- | --- | --- | --- |
| Ovarian | 8:8346690 | rs2976909 | hsa-miR-4660* | -0.2750 | 0.0987 | 0.0053 | 0.3963 |
| Ovarian | 20:60241152 | rs6061715 | hsa-miR-4758-5p | 0.3256 | 0.1423 | 0.0221 | NA |
| Ovarian | 4:110283708 | rs1859144 | hsa-miR-576-5p | 0.2646 | 0.1298 | 0.0414 | NA |
| SMR, summary data-based Mendelian randomisation; HEIDI, heterogeneity in dependent instruments; Chr, chromosome number; SNP, single nucleotide polymorphism; FDR, false discovery ratio, adjusted p-value; hsa, homo sapiens (human organism); miR, mature microRNA; 3p, 3-prime; 5p, 5-prime; NA reports if the number of SNPs used in the HEIDI analysis is smaller than 3. | | | | | | | |
