# Supplementary material for: Integrative Transcriptome-Wide Analyses Uncover Novel Risk-Associated MicroRNAs in Hormone-Dependent Cancers
Source: Front Genet. 2021 Aug 26;12:716236. doi: 10.3389/fgene.2021.716236 (PMC8427606; doi:10.3389/fgene.2021.716236)
Supplement: Supplementary file 5 [file Table_5.docx]

Table S5. SMR-HEIDI test results of colorectal cancer

| Cancer type | Chr: base pair position (top SNP) | rs ID (top SNP) | Associated miRNA | Effect Size | Standard Error | FDR (SMR) | P-value (HEIDI) |
| --- | --- | --- | --- | --- | --- | --- | --- |
| Colorectal | 17:27058296 | rs12948278 | hsa-miR-144-5p* | -0.2507 | 0.1001 | 0.0123 | NA |
| Colorectal | 17:26899502 | rs618791 | hsa-miR-144-3p | -0.2517 | 0.1018 | 0.0134 | NA |
| Colorectal | 7:158306735 | rs12534935 | hsa-miR-153-5p | -0.2174 | 0.0902 | 0.0159 | NA |
| Colorectal | 12:57886609 | rs2672571 | hsa-miR-1228-3p | -0.3323 | 0.1391 | 0.0169 | NA |
| Colorectal | 2:103066858 | rs11465730 | hsa-miR-4772-3p | 0.1803 | 0.0809 | 0.0259 | 0.3620 |
| Colorectal | 9:95373884 | rs1552908 | hsa-miR-3651 | 0.2026 | 0.0995 | 0.0417 | NA |
| Colorectal | 2:103034749 | rs4851581 | hsa-miR-4772-5p | 0.0803 | 0.0414 | 0.0426 | 0.2020 |

SMR, summary data-based Mendelian randomisation; HEIDI, heterogeneity in dependent instruments; Chr, chromosome number; SNP, single nucleotide polymorphism; FDR, false discovery ratio, adjusted p-value; hsa, homo sapiens (human organism); miR, mature microRNA; 3p, 3-prime; 5p, 5-prime; NA reports if the number of SNPs used in the HEIDI analysis is smaller than 3.
